# Non-communicable diseases in Saudi adolescents: prevalence, risk factors, and implications for public health

**DOI:** 10.3389/fpubh.2025.1542339

**Published:** 2025-02-06

**Authors:** Mansour Almuqbil, Syed Imam Rabbani, Rafiulla Gilkaramenthi, Mohammad Aljawadi, Walaa F. Alsanie, Abdulhakeem S. Alamri, Majid Alhomrani, Sara Alrouwaijeh, Amal F. Alshammary, Mohd Imran, Syed Mohammed Basheeruddin Asdaq

**Affiliations:** ^1^Department of Clinical Pharmacy, College of Pharmacy, King Saud University, Riyadh, Saudi Arabia; ^2^Department of Pharmacology and Toxicology, College of Pharmacy, Qassim University, Buraydah, Saudi Arabia; ^3^Department of Emergency Medical Services, College of Applied Sciences, AlMaarefa University, Riyadh, Saudi Arabia; ^4^Department of Clinical Laboratory Sciences, The Faculty of Applied Medical Sciences, Taif University, Taif, Saudi Arabia; ^5^Research Center for Health Sciences, Deanship of Graduate Studies and Scientific Research, Taif University, Taif, Saudi Arabia; ^6^Corporate of Pharmacy Services, King Saud University Medical City, Riyadh, Saudi Arabia; ^7^Department of Clinical Laboratory Sciences, College of Applied Medical Sciences, King Saud University, Riyadh, Saudi Arabia; ^8^Department of Pharmaceutical Chemistry, College of Pharmacy, Northern Border University, Rafha, Saudi Arabia; ^9^Department of Pharmacy Practice, College of Pharmacy, AlMaarefa University, Riyadh, Saudi Arabia; ^10^Research Center, Deanship of Scientific Research and Post-Graduate Studies, AlMaarefa University, Riyadh, Saudi Arabia

**Keywords:** non-communicable diseases, adolescents, disease prevalence, prevention, control

## Abstract

**Objectives:**

This study aimed to assess the prevalence of non-communicable diseases (NCDs) among Saudi adolescents, focusing on obesity, conduct disorder, asthma, and anxiety, and to identify potential risk factors associated with these conditions.

**Methods:**

A retrospective cross-sectional analysis was conducted using data from 2,160 adolescents sourced from official government databases and peer-reviewed literature. Statistical methods included one-way ANOVA, chi-square tests, logistic regression, and Pearson’s correlation coefficient, with significance set at *p* < 0.05 and a 95% confidence interval.

**Results:**

The prevalence of NCDs among Saudi adolescents was 11.8%. Obesity was the most common condition (odds ratio [OR] = 1.24, *p* = 0.006), followed by conduct disorder (OR = 1.12, *p* = 0.041), asthma (OR = 1.09, *p* = 0.036), and anxiety (OR = 1.06, *p* = 0.042). Pearson’s correlation revealed significant associations between these disorders and adolescence stages.

**Conclusion:**

Obesity, conduct disorder, asthma, and anxiety are significant health challenges for Saudi adolescents. These findings highlight the role of lifestyle factors such as diet and physical inactivity. Targeted interventions are needed to promote healthy behaviors and mitigate the long-term risks of these conditions.

## Introduction

Adolescents constitute a major proportion of the population worldwide. This stage of life is important since a child will be transforming into an adult. Both physiological and psychological changes take place during the phases of adolescence. These changes were found to be caused by the secretion of various hormones (1). Depending on the age, adolescence is categorized into early (11–13 years), middle (14–17 years) and late (18–20 years) stage (2). In the early stage, physiological growth and change start to occur in both males and females. The changes in the middle stage of adolescence include voice cracking in males, incidences of acne, and physical changes nearly complete in females and most have regular periods. Finally, in the late stage of adolescence, the modifications commonly observed are physical growth almost competes in both genders and are grown to full adult height (2, 3).

As reported in the literature, the adolescence stage of life could be associated with several diseased states. The important among them are behavioural alterations such as anxiety, depression, conduct disorders, agitation, and aggression (4). Besides, metabolic defects such as obesity, anorexia nervosa, and hyperlipidaemia were also found to be prevalent (3). Studies also indicated that diarrhoea, bronchial asthma, and *acne vulgaris* are seen during this stage of life (5). Determining the occurrences of common diseased states in the adolescent population was reported to assist in designing better proactive strategies and is one of the topics of research in the healthcare fraternity (6).

Saudi Arabia is a vast country with a population of 37,120,467, comprising 57% males and 43% females. According to a study, a major proportion of the population is the younger generation (7). The adolescents constitute about 20% of the total population of the country. The population of Saudi Arabia is distributed in urban as well as rural areas (8). The country after the discovery of petroleum oil has undergone a major economic transformation that has resulted in the migration of most people to cities and towns. This has resulted in a shift from agricultural-based occupations to non-agricultural activities (9). Further, an adaptation to Westernized food has increased the intake of unhealthy fast foods and colas, especially among the younger generations (10).

A study conducted on the adolescent population of Saudi Arabia indicated that about 25% of children are obese. One of the reasons attributed to this is unhealthy diet habits and physical inactivity (11). Childhood obesity according to a study is the gateway for several metabolic diseases such as diabetes mellitus, hyperlipidaemia, hypertension, and atherosclerosis (12). In this context, it was predicted that by 2050, Saudi Arabia could become the major sufferer of diabetes mellitus among its population in the region (13). Some studies conducted in the past reported the prevalence of non-communicable diseases (NCDs) in the population of Saudi Arabia (14, 15). However, the prevalence in different stages of adolescence was not studied extensively in the literature. Additionally, the country has a significantly large population of adolescents aged public (13), which emphasizes determining the prevalence of NCDs in this group. The present study aimed to assess the prevalence and significance of non-communicable diseases (NCDs) among the adolescent population in Saudi Arabia. The findings are intended to offer valuable insights that can inform the development of proactive strategies for preventing the onset of these diseases within this population.

## Methods

### Study design

A cross-sectional retrospective study was conducted between September 2022 and August 2023. Official and published information from the Ministry of Health, Saudi Arabia was retrieved from the websites such as Ministry of Health, Saudi Arabia^1^^,^^2^^,^^3^ and raw data about the prevalence of various diseases in the population was obtained from Centers for Disease Control and Prevention.^4^ The published data about the types of non-communicable diseases in the scientific articles were also retrieved for determining the prevalence in adolescents (14, 15). Epidemiological data such as population size, age, gender, family status, and place of residence was collected from the General Authority of Statistics, Kingdom of Saudi Arabia.^5^

### Study setting

Data from various sources, including official websites and scientific journals, were retrieved and compared to assess their impact, as reported in the literature. Saudi Arabia, a vast country with both urban and rural areas, consists of different provinces that experience significant climatic variations. Additionally, non-communicable diseases have been shown to be influenced by a range of socio-economic and psycho-social factors (16). To ensure thoroughness, the study was carefully designed to span a full year, with prevalence data collected from various official websites on a month-wise basis. Efforts were made to collect data from all seasons to account for seasonal variations, using information published on official websites. Given the study’s broad geographical scope, the research team took measures to ensure that data from all regions of the country were included. Any discrepancies encountered during data collection, interpretation, analysis, or potential biases were addressed through discussions among the research team.

### Inclusive and exclusive criteria

To achieve a uniform and logical availability of data, the inclusive and exclusive criteria were followed. These criteria are the information published in the English language with clarity on age, gender, schooling, parents’ details, type of residency, and prevalence of diseased states. Information other than English language, incomplete details, and inadequate representation of data in the studies were excluded from the study. Insufficient data about the characteristics of non-communicable diseases in the population including the demographic nature was also excluded from the study (17).

### Ethical consideration

This study aims to present information from various online sources in a scientific manner, benefiting the public and supporting further research in the emerging field of microbe-related diseases. The study adhered to established guidelines and procedures outlined in the existing literature (18) while gathering data. Information from public sources, including official websites and published scientific papers, was carefully collected, with strict attention to maintaining privacy for individuals, groups, and organizations.

The research relied on publicly accessible data, including open, reusable, and redistributable datasets that had no usage restrictions. No restricted, censored, or protected information was included in the study. Data that was controversial, incomplete, lacked ethical approval, or used inappropriate statistical methods was excluded. All resources utilized in the data collection are properly cited in the manuscript. Efforts were made to minimize bias and errors by blinding the study protocol and applying the Newcastle-Ottawa scale. Standard statistical methods, commonly used in literature, were employed to further minimize the risk of errors during data analysis.

### Quality assessment

To evaluate the risk of bias in cross-sectional studies, the Newcastle-Ottawa Scale (NOS) was applied. This tool assesses various domains, with a particular focus on the sampling strategy, statistical analysis methods, and study outcomes (19). The quality of the studies was assessed in a blind manner by the authors, who assigned codes to the data collected from different regions and seasons. Besides, the data was anonymously analyzed, and the results were discussed only in the blinded outcome evaluation meetings. Furthermore, any discrepancies that arose during such meetings were addressed through discussions with a subject expert. Studies included in the analysis were required to score above 3 on the Newcastle-Ottawa Scale as part of the eligibility criteria. Of the 108 articles retrieved from the literature, 17 met these criteria and were included in the analysis.

### Descriptive analysis

The data collected from the authenticated sources was entered in the MS Excel sheet. A record of the prevalence of common non-communicable diseases was noted down in different months and its percentage prevalence was calculated. Only the documented information from authentic resources was recorded for the study. The important parameters of descriptive analysis are (20–22).

- Demographic characteristics – the study population was segregated according to gender, stage of adolescence, place of residency, schooling type, and parents’ details.
- Prevalence of diseased states – in the study population of adolescents, the prevalence of several comorbid conditions recorded was noted. The data was further analyzed to indicate ‘no disease’, ‘one disease’ and ‘two or more diseases’.
- Stages of adolescence – the study population was categorized into three stages depending on age, such as;

 ■Early stage: 11–14 years
 ■Middle stage: 15–17 years
 ■Late stage: 18–20 years

The prevalence of common non-communicable diseases in adolescents was determined based on the literature (23) and subsequently redistributed according to the stages of adolescence (24). Data analysis was conducted to calculate the percentage prevalence of each disease at each stage of adolescence.

- Correlation analysis – the data recorded for the prevalence of different non-communicable diseases was subjected to correlation analysis to determine the likelihood of these diseases in the adolescent population.

### Representation of data

**Figure 1:**
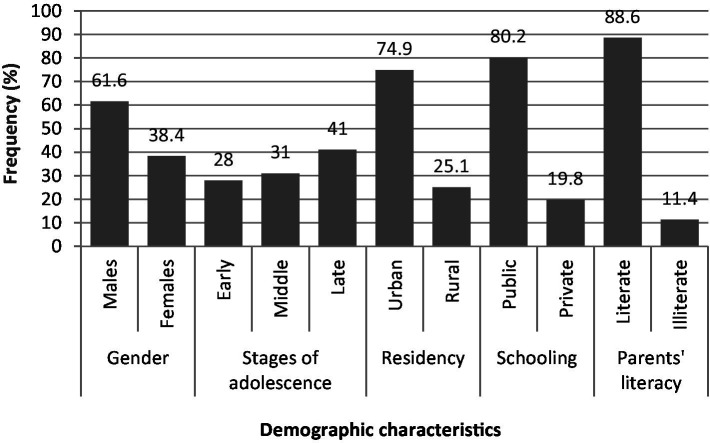
Demographic characteristics of adolescents. The values are represented as percentage frequency in the study population.

**Figure 2:**
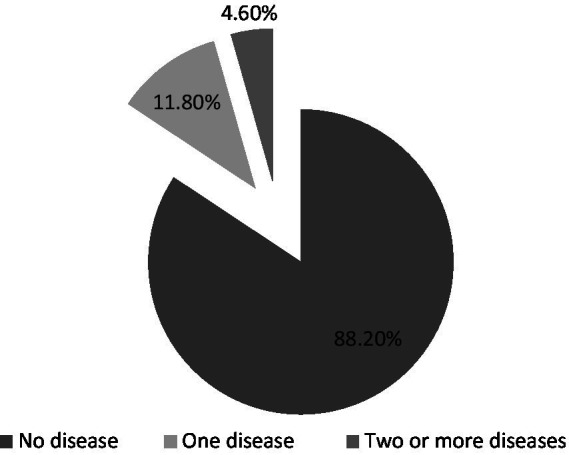
Frequency of diseases in adolescents. The values are represented as percentage frequency in the study population.

**Figure 3:**
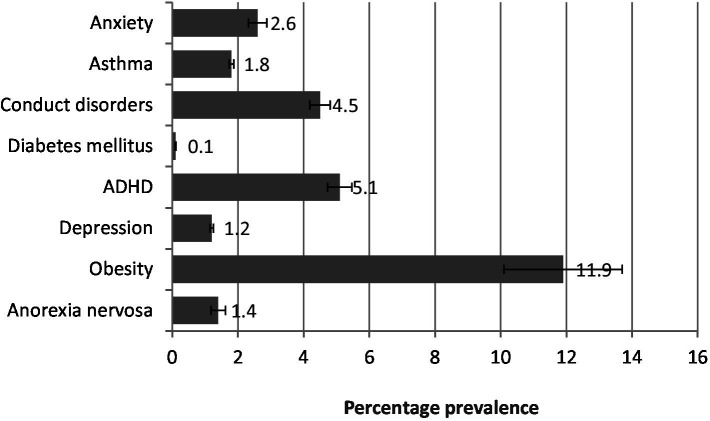
Prevalence of diseases in the early stage of adolescence. The values are represented as percentage mean ± SEM.

**Figure 4:**
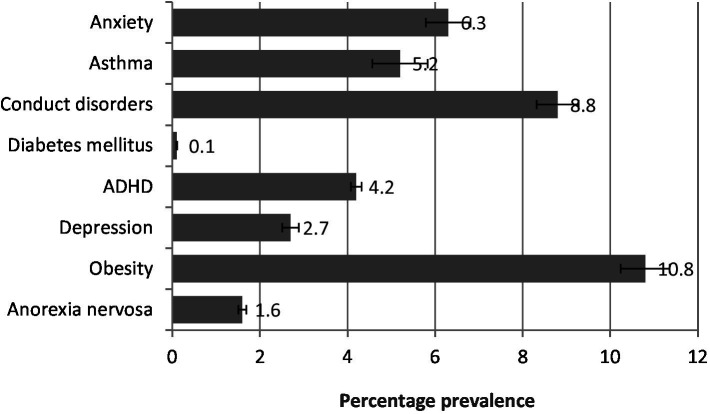
Prevalence of diseases in the middle stage of adolescence. The values are represented as percentage mean ± SEM.

**Figure 5:**
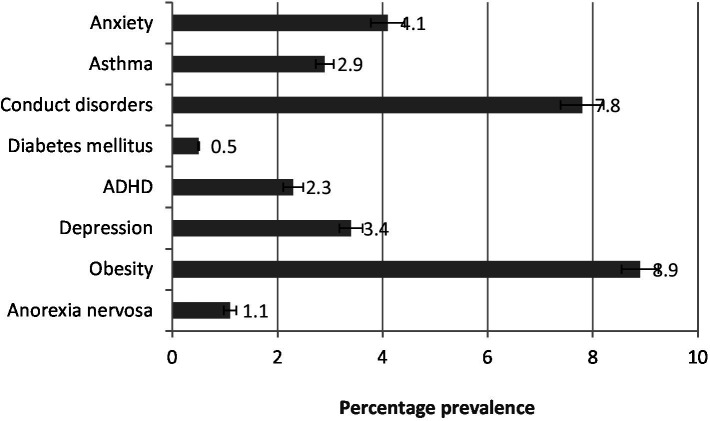
Prevalence of diseases in the late stage of adolescence. The values are represented as percentage mean ± SEM.

**Figure 6:**
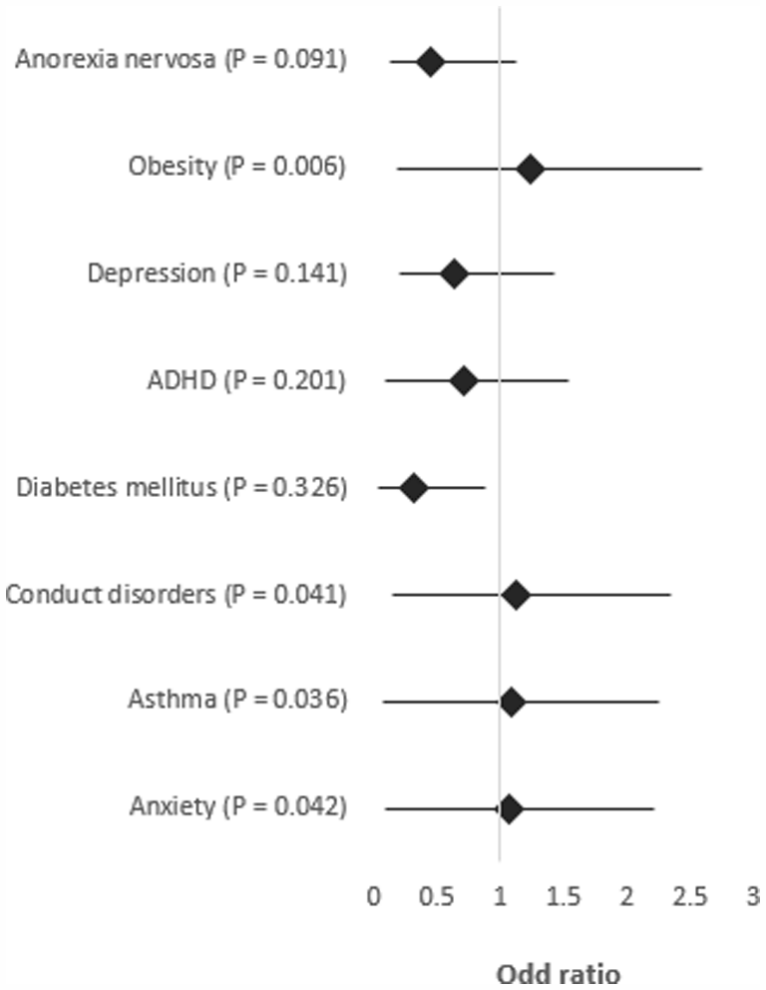
Forest plot of logistic linear regression analysis. One-way ANOVA and chi-square test. *p* less than 0.05 was considered significant.

The data collected from the population was analyzed and represented in figures (19). The demographic characteristics of the study population are indicated in Figure 1. The prevalence of all diseased states in the study population is represented in Figure 2. The prevalence of non-communicable diseases in the three stages of adolescence such as early, middle, and late are summarized in Figures 3 –5, respectively. Finally, the correlation between the prevalence of non-communicable diseases in the study population is shown in Figure 6 and Table 1.

**Table 1:** Determinants of NCDs in the adolescents’ population of Saudi Arabia.

Types of NCDs	Pearson correlation coefficient (*r*-value)		
	Early stage	Middle stage	Late stage
Anorexia nervosa	0.09	0.17	0.08
Obesity	0.72**	0.86**	0.81**
Depression	0.01	0.06	0.12
ADHD	0.38*	0.19	0.07
Diabetes mellitus	0.04	0.08	0.16
Conduct disorders	0.32*	0.41*	0.34*
Asthma	0.14	0.30*	0.44*
Anxiety	0.10	0.31*	0.39*

Statistics: Pearson’s correlation analysis. Data is representative of coefficient value (*r*). *p < 0.05, **p < 0.01 compared between groups.

### Ethical clearance

The study received ethical approval from the Institutional Review Board of AlMaarefa University, Riyadh, Saudi Arabia (IRB22-026) for conducting scientific analysis on data collected from various electronic resources.

### Statistical analysis

The data obtained from the study was statistically analyzed by SPSS IBM 25 software. The data collected from 2,160 adolescents were categorized into different age groups. Incomplete data of about 72 adolescents was not considered due to the requirements of the statistical software. In addition, this number was excluded from the 2,160 adolescents’ records that were used for the analysis. The data for each group of population was analyzed for percentage prevalence. One-way analysis of variance (ANOVA) was used to test the level of significance, followed by a chi-square test to determine the association between the occurrences of non-communicable diseases in the study population. Furthermore, the relationship between different stages of adolescents and non-communicable diseases was ascertained using the Pearson correlation coefficient. A fixed value of confidence interval (lower – 95% and upper – 95%) was kept while analyzing the data (19, 22). *p-values* less than 0.05 were considered to determine the significance of all analyses.

## Results

### Demographic characteristics

Figure 1 presents the demographic characteristics of the adolescent population retrieved from different resources: 61% are male, 41% are in late adolescence (18–20 years), 75% live in urban areas, 80% attend public schools, and 88% have literate parents.

### Frequency distribution of non-communicable diseases in the study population

According to the data collected, 88.2% of adolescents are free from non-communicable diseases, while 11.8% have at least one, and 4.6% suffer from two or more (Figure 2).

### Prevalence of diseases in the early stage of adolescence

As per the records represented in Figure 3, the prevalence of key health conditions in early adolescence: obesity (11.9%) was most common, followed by ADHD (5.1%), conduct disorders (4.5%), anorexia nervosa (1.4%), anxiety (2.6%), asthma (1.8%), depression (1.2%), and diabetes mellitus (0.1%).

### Prevalence of diseases in the middle stage of adolescence

Figure 4 shows that in the middle stage of adolescence, obesity (10.8%) is the most common disorder, followed by conduct disorder (8.8%), anxiety (6.3%), asthma (5.2%), ADHD (4.2%), depression (2.7%), anorexia nervosa (1.6%), and diabetes mellitus (0.1%).

### Prevalence of diseases in the late stage of adolescence

Figure 5 shows that in late adolescence, obesity (8.9%) is the most prevalent disorder, followed by conduct disorder (7.8%), anxiety (4.1%), depression (3.4%), asthma (2.9%), ADHD (2.3%), and anorexia nervosa (1.1%). Diabetes mellitus increased slightly to 0.5% but remains the least prevalent condition.

### Summary of correlation analysis

The correlation analysis indicated that obesity had the highest adjusted odds ratio (OR = 1.24, CI = 1.35/1.06, *p* = 0.006), followed by conduct disorders (OR = 1.12, CI = 1.22/0.98, *p* = 0.041), asthma (OR = 1.09, CI = 1.17/1.02, *p* = 0.036), and anxiety (OR = 1.06, CI = 1.16/0.96, *p* = 0.042). Diabetes mellitus had the lowest odds ratio (OR = 0.32, *p* > 0.05), and anorexia nervosa, depression, and ADHD showed non-significant *p*-values (>0.05) (Figure 6).

Pearson’s correlation analysis revealed significant associations between stages of adolescence and non-communicable diseases. In early adolescence, significant correlations were found for conduct disorders (*p* < 0.05) and obesity (*p* < 0.01). In middle adolescence, significant correlations were observed for anxiety (*p* < 0.05), asthma (*p* < 0.05), conduct disorders (*p* < 0.05), and obesity (*p* < 0.01). The same four diseases showed significant correlations in late adolescence at similar significance levels (Table 1).

## Discussion

The present study revealed that 11.8% of the adolescent population in Saudi Arabia suffers from non-communicable diseases (NCDs). Obesity was the most prevalent condition across all stages of adolescence, followed by conduct disorder, which was most common in middle and late adolescence. Attention deficit hyperactivity disorder (ADHD) was more frequently observed in early adolescence. Additionally, asthma and anxiety were also prominent, supported by correlation analysis (Figures 1–6; Table 1).

Obesity has emerged as one of the most common disorders among adolescents, particularly in developed countries. The findings of this study are consistent with previous research conducted in the Eastern Province of Saudi Arabia, which reported a 1.25-fold increase in adolescent obesity (25). Similar trends have been observed globally, where the prevalence of obesity among children and adolescents is rising at an alarming rate (26). This increase is paralleled by a corresponding rise in diabetes mellitus among the Saudi population (27). Obesity is a well-established risk factor for diabetes mellitus and serves as an early marker for other metabolic diseases such as hypertension, myocardial infarction, and stroke (28).

Children with conduct disorder often exhibit aggression and a disregard for others. Several factors, including genetic, organic, and biochemical influences, have been linked to this disorder (29). Studies indicate that such aggressive behavior is more common among individuals living in urban areas. Contributing risk factors include nuclear family structures, dysfunctional families, substance abuse, psychosocial issues, and traumatic experiences (30). Additionally, there has been a significant global rise in mental illness, with depression, anxiety, and stress being major contributors (31). Parental mental illness is also reported to contribute to the incidence of conduct disorders in children (32).

The analysis also revealed a likelihood of bronchial asthma in the population, consistent with previous research that highlighted higher prevalence of asthma, particularly in children (33). Risk factors for asthma include tobacco smoking, environmental pollution, stress, and obesity. Seasonal variations and climatic conditions, such as dust and sandstorms in Saudi Arabia, further exacerbate the condition (34). A past study suggested that individuals living in areas prone to such environmental conditions are more susceptible to respiratory diseases, including asthma (35). The increasing prevalence of cigarette smoking among adolescents, some of whom start as early as 15 years old, has become a significant public health concern (36, 37).

Anxiety disorders were also found to more in the study population. This finding supports earlier research that identified childhood anxiety as a common mood disorder among adolescents (38). Like conduct disorder, anxiety can be triggered by stress, traumatic experiences, biochemical variations in the brain, and genetic predisposition (29, 30). Childhood anxiety is associated with symptoms such as restlessness, poor eating and sleeping habits, difficulty concentrating, and a tendency to become easily angered or aggressive (4, 29). Previous research has linked conduct disorder with other mental illnesses, such as depression, anxiety, and ADHD (31), which was also observed in this study across different stages of adolescence.

The consumption of junk foods by adolescents are known to contain high in fats, sugars, and additives, has been associated with various health issues, including obesity, anxiety, depression, asthma, and conduct disorder symptoms (39, 40). Pearson’s correlation coefficient analysis also indicated the relationship between these non-communicable diseases with adolescence in the study population. Maintaining a healthy lifestyle through regular physical activity, a nutritious diet, consistent sleep schedules, and the avoidance of addictive substances like nicotine are traditional practices for sustaining good health (41).

### Implications and limitations of the study

The findings from this study provide preliminary insights into the prevalence of NCDs among Saudi Arabian adolescents. Healthcare providers can use these insights to design strategies aimed at preventing or reducing the occurrence of NCDs in the population. Customized preventive or therapeutic approaches, aligned with international and World Health Organization guidelines, could be directed towards specific demographic groups categorized according to the stages of adolescence (41). Awareness programs about adolescent diseases at different stages, and their impact on later stages of life, can be designed to educate the public.

However, the study’s reliance on retrospective data from a fixed population introduces potential bias, limiting the generalizability of the findings. The data may not fully represent the entire adolescent population of Saudi Arabia. Future research should involve larger, more diverse populations from different regions of the country to support these findings. Including the influence of other factors, such as familial, socioeconomic, environmental, and ethnic background, could provide additional insights for the research. Moreover, adopting randomization techniques and utilizing other statistical analysis tools to minimize bias during the literature retrieval process would significantly enhance the authenticity of the findings.

## Conclusion

This retrospective study of an adolescent population in Saudi Arabia identified obesity, conduct disorder, asthma, and anxiety as the most common NCDs. These findings align with previous research on the prevalence of these conditions in young populations. Considering the increasing prevalence of metabolic diseases such as diabetes mellitus, the data from this study could be valuable for healthcare providers. New proactive strategies are needed to reduce the frequency of these conditions among the younger generation.

Given the harsh climatic conditions in many desert regions of the country, indoor sports activities can be promoted for students. Additionally, awareness programs in schools and communities should highlight the hazards of unhealthy lifestyle and offer methods for managing various types of stress. Further research is needed to substantiate these findings through more extensive retrospective and prospective studies, with a focus on identifying the causes, diagnosis, and management of obesity, conduct disorders, asthma, and anxiety in this population.

## Data Availability

The original contributions presented in the study are included in the article/supplementary material, further inquiries can be directed to the corresponding author.

## Footnotes

^1^ https://www.moh.gov.sa/en/Pages/default.aspx

^2^ https://www.moh.gov.sa/en/Ministry/Statistics/book/Documents/1433.pdf

^3^ https://www.moh.gov.sa/en/Ministry/Statistics/Indicator/Pages/Indicator-1440.aspx

^4^ https://www.cdc.gov/globalhealth/countries/saudi_arabia/default.htm

^5^https://www.stats.gov.sa/en, https://moe.gov.sa/en/Pages/default.aspx
